# Supplementary material for: Are carbon emissions trading and green financial instruments synergistic? -Comprehensive quantitative research based on content analysis
Source: PLoS One. 2024 Mar 7;19(3):e0298601. doi: 10.1371/journal.pone.0298601 (PMC10919601; doi:10.1371/journal.pone.0298601)
Supplement: S1 Data — (ZIP) [file pone.0298601.s001.zip › Data/地级市Z_F工作报告.zip!/╔╜╢1⁄2/╟α╡║╩╨/└┤╫╘╣1⁄2╓┌║┼í╛┬φ┐╦╩2╛▌═°í┐_2006.docx]

各位代表：

　　现在,我代表市人民政府向大会作工作报告,请连同《青岛市国民经济和社会发展第十一个五年规划纲要(草案)》一并审议,并请各位政协委员和其他列席人员提出意见。

　　一、“十五”时期国民经济和社会发展回顾

　　“十五”时期的五年,是充满机遇和挑战的五年,是青岛经济社会发展最好的历史时期之一。在党中央、国务院,省委、省政府和市委的领导下,我们紧紧依靠全市人民,坚持以邓小平理论和“三个代表”重要思想为指导,全面贯彻落实科学发展观,抓住国家宏观调控、加入世贸组织、迎办奥运等重大机遇,全力推进“三件大事”,繁荣青岛、平安青岛、文明青岛建设取得巨大成就,“十五”计划主要目标提前完成,实现了青岛发展的历史性跨越。

　　(一)经济社会全面发展,城市核心竞争力显著提升。五年来,我们紧紧抓住发展这个第一要务,坚持以经济建设为中心,努力推动国民经济持续快速协调健康发展。与2000年相比,预计全市生产总值、人均生产总值和地方财政收入均实现翻番。郊区五市经济发展迅速,总量已占全市的50%左右。城市化水平达到58.5%。全面实施挺进西海岸战略,新的经济重心基本形成。物质文明、政治文明、精神文明、和谐社会建设同步推进,进入首批全国文明城市行列。荣获“CCTV中国最具经济活力城市”称号。成功获得2008北京奥运会帆船比赛承办权。圆满承办亚洲合作对话第三次外长会议、中国国际消费电子博览会、APEC中小企业技术交流暨展览会等一系列重大活动,进一步提高了城市的知名度和影响力。

　　(二)结构调整实现突破,可持续发展经济体系初步形成。五年来,坚持以结构调整为主线,加快产业优化升级步伐,不断提高经济增长的质量和效益,产业竞争力进一步增强。国民经济三次产业比例由12.2∶48.7∶39.1调整到6.7∶51.9∶41.4,轻重工业比例由64.1∶35.9调整到51∶49。高新技术产业产值占工业总产值比重达到43%。特色经济加速发展,集装箱吞吐量和旅游总收入分别增长2倍和1.5倍,海洋产业增加值达到300亿元。家电、电子、汽车、造船、石化、港口等一批带动产业结构升级的重大项目相继开工,“国字号、大企业、多品牌”的产业格局开始形成,六大产业集群占全市工业比重达到40%。金融、物流、会展等现代服务业发展加快,第三产业增加值年均增长14%以上。万元生产总值能耗由1.1吨标准煤降低到0.9吨标准煤。

　　(三)城乡统筹互动发展,现代化城市大框架基本确立。五年来,坚持高起点规划、高水平建设、高效能管理,“三点布局、一线展开、组团发展”的大城市发展战略得到全面实施,加快了城乡一体化进程。城市总体规划修编、生态市规划、滨海公路沿线组团总体规划等编制完成。基础设施建设累计投资720亿元,比“九五”时期增长2.1倍。同三高速青岛段、流亭机场扩建一期、东西快速路等一批重点交通工程交付使用,滨海公路、海湾大桥、胶济铁路电气化改造青岛段、国际帆船中心等一批城市功能项目开工建设,滨海步行道市区段、汇泉广场改造、崂山巨峰、海底世界等一批旅游特色项目相继竣工,重点河道综合治理、小街小巷整治等一批民生环境工程陆续完成。加大了旧城改造和市区北部基础设施的建设力度,城区面貌不断改善。全面启动了生态市建设,五市三区全部建成国家级生态示范区,前海一线截污、胶州湾环境保护等工程加快实施,生态环境质量明显提高,顺利通过国家环保模范城的复检。获得“中国人居环境奖”。进一步理顺城市管理体制,“两级政府、三级管理、四级网络”的管理格局基本形成。

　　各级政府把解决“三农”问题作为各项工作的重中之重,坚持统筹城乡“七个互动”,推进工业基地和产业集群向郊区布局、城市基础设施向郊区延伸、城市公共服务向郊区覆盖,促进了农村经济社会的全面快速发展。五年来,各级财政“三农”支出累计达到98.9亿元,其中市本级29.9亿元。郊区建成一批工业生产和配套基地,农村服务业发展和小城镇建设成效显著,加速了农村工业化、城市化进程。农业结构调整和产业化步伐加快,外向型农业加速发展,畜牧、水产业占农业比重分别提高了4.8和7个百分点。农村基础设施建设得到加强,83%的村庄通了柏油路,自来水普及率提高到82%。全面落实种粮、良种和农机具购置补贴等各项惠农政策,全面取消了农业税征收,历史上首次实现了农民“零负担”。取消了农民进城务工的限制性规定和不合理收费,培训、转移农村劳动力11万人和28万人。“以县为主”的农村义务教育管理体制进一步完善。农村社会养老保险制度稳步推开,新型农村合作医疗人口参保率达到91.7%。

　　(四)改革开放步伐加快,发展活力持续增强。五年来,坚持以市场化为导向,以体制创新为重点,不失时机地推进了各项改革。国有经济布局和战略调整取得成效,大企业引进战略投资伙伴和产权多元化实现突破,全市主要经济系统国有企业改制面达到90%以上。新型国有资产管理体制框架基本建立,国有资产监管得到加强。加大对民营经济的引导和扶持,制定实施了加快发展的政策措施,民营经济占全市比重达到44%。全面推进政府管理体系“五项工程”,坚持依法行政,实行科学民主决策,政府职能逐步转变,社会管理和公共服务职能进一步加强。顺利完成两轮政府机构改革,市级行政许可事项削减72.4%。深化财税体制改革,建立了市与区市财源建设联动机制。努力提高对外开放层次和水平,招商引资和对外贸易迈上新台阶。五年累计实际利用外资151亿美元,利用内资780亿元,分别是“九五”时期的3.1倍和6.5倍。丹麦马士基、韩国GS、日本东丽、中石化、中船重工、一汽、中海油、中国铝业等一批中外大企业来青投资。出口加工区、保税物流园区先后封关运营。2005年进出口总额完成306亿美元,是2000年的2.8倍。实施“走出去”战略取得较大进展。外事、侨务、对台、口岸工作进一步加强。与国外23个城市缔结友好城市关系或友好合作城市关系。积极推进了对口支援工作。

　　(五)社会事业全面进步,文明城市彰显魅力。五年来,坚持协调推进经济社会发展,社会主义精神文明建设和民主法制建设得到加强,各项社会事业呈现繁荣发展的崭新局面。城市创新体系日益完善,五年获得国家科学技术奖21项。以海尔、海信等为代表的企业自主研发体系初步形成。加强知识产权保护,专利授权量增长74%。全市研发经费占生产总值的比重达到2.2%,提高1.04个百分点。国家深海潜水器基地定点我市,国家海洋科学研究中心开始筹建。完成义务教育标准化工程。初中毕业生升学率达90%以上,普、职高中协调发展,高等院校全日制普通在校生比2000年增加4倍。中国石油大学等一批高校在我市建校办学。扩建改造了市图书馆、群众艺术馆、美术馆等一批公共文化服务设施,开工建设了凤凰岛影视文化基地、青岛大剧院、极地海洋世界、现代艺术中心、市体育馆等一批重点文化工程。有线数字电视在全国率先实现整体转换。获得国家级各类文艺奖项294个。国防动员、人民防空和民兵预备役建设取得新成效,第五次获得“全国双拥模范城”。城乡公共卫生和医疗服务体系不断完善。人口与计划生育综合改革全面推进,低生育水平保持稳定。建成了老年大学、老年公寓等一批服务设施。第一次经济普查工作圆满完成。新闻出版、民族、宗教、档案、史志、仲裁、妇女、儿童、残疾人等社会事业全面发展。

　　各级政府认真执行人大及其常委会的决议决定,坚持重大事项报告制度,自觉接受法律监督和工作监督。坚持重大问题与人民政协协商制度,自觉接受民主监督。五年来,提请市人大常委会审议通过地方性法规34件,制定政府规章69件,办复市人大代表议案、建议和政协提案、建议案6899件。基层民主进一步扩大,依法治市稳步推进。广泛开展“双学三创”活动,“诚信、和谐、博大、卓越”的城市精神深入人心,涌现出许振超等一批典型代表。连续3年开展了“我为青岛发展献计策”市民月活动。全市注册志愿者达33.5万人。

　　(六)居民生活持续改善,社会环境和谐稳定。五年来,坚持以人为本、以民生为重,适时调整了关系民生的就业、保障、住房、收入等政策,集中力量为人民群众办实事,切实维护广大人民群众的根本利益。市场导向的就业机制和困难群体就业帮扶机制基本建立,累计实现城镇就业53万人,登记失业率控制在4%以内。市本级社会保障性支出占财政预算支出的比重由2000年的7.2%提高到9.4%。企业养老保险参保人数净增33万人,退休人员养老金水平提高38%,城乡居民最低生活保障标准分别提高了30%和80%。推进经济适用住房、普通商品住房等多层次住房供应体系建设,解决了市区人均使用面积6平方米以下低保家庭的住房困难。城市居民人均可支配收入增长61.2%,农民人均纯收入增长59.5%。国有、集体企业拖欠职工工资和建设领域拖欠农民工工资问题得到较好解决。进一步完善了价格决策听证制度,物价水平保持基本稳定。扎实开展“平安青岛”创建活动,大力加强基层基础工作,确保了政治安全、社会安全、信访安全和生产安全。刑事案件、治安案件、集体上访和安全生产事故发生起数整体下降,荣获“全国社会治安综合治理先进城市”称号。适应公共管理的新需要,建立了自然灾害、事故灾难、公共卫生、社会安全等方面64个专项应急预案和分预案,公共安全应急体系初步形成。加强食品药品安全管理,被命名为全国“三绿工程”样板城市。

　　刚刚过去的2005年,是“十五”时期青岛经济社会发展最快最好的一年。与上年相比,预计全市生产总值增长16%以上。地方财政一般预算收入增长35.1%。全社会固定资产投资增长40%以上。社会消费品零售总额增长15%。城市居民人均可支配收入和农民人均纯收入达到12920元、5800元,分别增长16.5%和14%,均为1999年以来的最高水平。年初确定的40个产业集群项目已开工和竣工37个,占92.5%。以企业为主体的自主核心技术研发和创造国际技术标准取得突破。在全国首批3个中国世界名牌产品中占据2席,荣膺“中国品牌之都”。引进8家中外金融机构。年初确定重点办好的12件实事顺利完成或开工建设。成功举办中国国际小提琴比赛、国际马拉松比赛和青岛市第一届城市运动会。圆满完成中俄联合军事演习和多项国家重要活动的服务保障任务。开展了城市民兵防空演练活动。有效抵御台风影响,实现了安全度汛。全力抓好高致病性禽流感防控工作,确保了全市无重大疫情。深入开展了保持共产党员先进性教育活动,基层组织得到加强,机关作风得到改善。在创建全国文明城市活动中,全市人民广泛参与,收到了显著成效。

　　各位代表!过去的五年,在市委的正确领导下,我们全面实施一系列重大发展战略,抢抓机遇,与时俱进,全市人民解放思想、干事创业、加快发展的氛围更加浓厚;我们坚持以改革开放促发展,努力开拓,锐意进取,经济社会各个领域敢于创新、勇于争先的气势空前高涨;我们牢固树立科学发展观,坚决贯彻中央宏观调控各项措施,全市上下注重以人为本、统筹发展的理念大大增强;我们经受住了SARS疫情等的严峻考验,积极推进和谐社会建设的伟大实践,各级政府依法行政,解决突出矛盾和应对突发事件的能力显著提高。“十五”时期所取得的巨大成就,揭开了新青岛新世纪新发展的辉煌一页,为在新的起点上实现新跨越奠定了坚实基础。在这里,我代表市人民政府,向为青岛发展做出贡献的历届市领导班子的各位老同志致以崇高的敬意!向辛勤工作在各条战线的全市人民,向各民主党派、工商联、人民团体和社会各界,向中央、省驻青单位,向人民解放军和武警驻青部队,向所有来青的投资者和劳动者,向关心支持青岛发展的海内外朋友们表示衷心的感谢!

　　各位代表!当前我市在加快发展中还面临许多困难和矛盾,政府工作还存在一些不足和差距。经济结构调整和增长方式转变任务艰巨,自主创新能力亟待提升,民营经济实力不够强,现代服务业比重偏低,消费拉动相对较弱。城市管理存在薄弱环节,综合服务功能有待全面加强。城乡发展不够平衡,解决“三农”问题需要下更大的气力。资源和环境支撑能力较弱,高层次和高技能人才短缺逐渐显现,体制改革难度加大。政府自身建设和职能转变还需加大力度,投资发展环境有待进一步改善。对此,我们要始终保持清醒的头脑,增强忧患意识和责任感,采取更加有力措施,认真加以解决。

　　二、“十一五”时期的指导方针和目标任务

　　“十一五”时期,是全面建设小康社会的关键时期,是推动我市经济建设、政治建设、文化建设、社会建设实现新跨越,促进青岛更加繁荣富裕、更加文明进步、更加稳定和谐的战略机遇期。站在新的历史起点,面对光荣的使命和艰巨的任务,我们必须以科学发展观为指导,按照“继承、转变、改革、提升”的要求,继续深入推进“三件大事”,把握好新一轮大发展的“十个重点”,切实把经济社会发展转入科学发展的轨道。《纲要(草案)》提出“十一五”时期全市国民经济和社会发展的指导方针是:以邓小平理论和“三个代表”重要思想为指导,坚持以科学发展观统领经济社会发展全局,全面贯彻党的十六大和十六届三中、四中、五中全会精神,按照省委、省政府关于发挥青岛龙头带动作用的要求、市九次党代会以来确定的一系列战略部署,围绕建设全国重点中心城市、世界知名特色城市的奋斗目标,把提高人民群众的生活水平和质量、促进人的全面发展作为经济社会发展的根本出发点和落脚点,进一步深化改革、扩大开放,切实转变经济增长方式,创新发展模式,建设节约型社会,加速融入环渤海经济圈,积极发挥对山东半岛城市群的辐射带动作用,推进国民经济持续快速协调健康发展和社会全面进步,努力构建社会主义和谐社会。

　　“十一五”时期,预期全市生产总值年均增长13%,地方财政收入与国民经济保持同步增长,城市居民人均可支配收入和农民人均纯收入年均分别增长10%左右。经过“十一五”的发展,全面建设小康社会将取得更大成就,综合经济实力、国际竞争力和城市文明程度将提高到一个新水平,人民生活水平和质量将再上一个新台阶。

　　根据上述指导方针和目标,着力抓好六个方面的工作:

　　(一)加快转变经济增长方式,努力实现可持续发展。切实把增强自主创新能力作为科学技术发展的战略基点和调整产业结构、转变增长方式的中心环节,更多地依靠自主创新和提高劳动者素质推动经济发展。集中优势资源实施“五个重点”攻关,开发并掌握一批核心技术和关键技术,初步形成以市场为导向、企业为主体、产学研相结合的自主创新体系。坚持以自主创新提升品牌经济竞争力,培育一批拥有自主知识产权、知名品牌和较强国际竞争力的优势企业。加快发展循环经济,推动重点行业、领域、园区的清洁生产、集约化发展,努力建成全国循环经济示范城市。全面落实节约资源的基本国策,加强土地和海洋资源管理,提高资源综合利用能力。健全环境监管体制,强化重点污染源防治和生态环境保护,不断改善环境质量,建设资源节约型、环境友好型社会。

　　(二)推进产业结构优化升级,构筑现代产业体系。“十一五”期间,形成新型大工业主导、现代服务业支撑、农业产业化水平不断提高的产业结构。围绕已确定的重大产业项目,积极推进三大特色经济、四大工业基地和六大产业集群建设,壮大电子信息、新材料和生物技术等高新技术产业,以现代技术改造食品饮料、纺织服装、化工橡胶、机械加工等传统优势产业,加快培育以产业装备、零部件制造装备、测试装备为重点的装备制造业,基本建成现代制造业基地。继续实施“以港兴市”战略,建设国际集装箱枢纽港,大力推进金融保险、旅游会展、信息咨询、总部经济、创意产业等现代服务业,繁荣商贸服务业,促进先进制造业与现代服务业协调发展,努力建成航运、物流、商贸中心和金融、信息、中介服务中心。进一步提高农业科技水平,以产业化、标准化、市场化、国际化为抓手,大力发展高产、优质、高效、生态、安全的现代农业。

　　(三)深入实施城乡互动战略,加快建设社会主义新农村。按照“生产发展、生活宽裕、乡风文明、村容整洁、管理民主”的要求,促进郊区经济社会全面发展。坚持“工业反哺农业、城市支持农村”和“多予少取放活”的方针,加大对农业和农村投入力度,扩大公共财政覆盖农村的范围,建立以工促农、以城带乡的长效机制。加快推进郊区新型工业化,构筑区域化布局、产业化经营和社会化服务的现代农业新格局,促进郊区经济转型升级。加强农村富余劳动力技能培训和农民工岗位培训,引导富余劳动力向非农产业和城镇有序转移,千方百计增加农民收入。继续深化农村改革,基本完成乡镇机构、农村义务教育和县乡财政管理体制改革任务。强化政府对农村的公共服务,围绕“水、电、路、医、学”等方面,加大对农业和农村基础设施建设投入力度,加快发展农村公共事业。进一步优化农村中小学布局,促进城乡教育均衡发展。全面落实农村义务教育“两免一补”政策。完善新型农村合作医疗制度,稳步推进新型农村社会养老保险制度。积极建设农村文化设施和文化活动场所,着力发展农村特色文化,不断提高农民整体素质和农村文明程度。搞好农村社区规划,全面开展村庄整理和环境整治,明显改善农村的生产生活条件和整体面貌。加大对口扶贫工作力度,基本解决库区移民村生产生活条件差和贫困落后问题。

　　(四)促进区域协调发展,提高城市现代化水平。坚持城市规划建设“七项原则”,按照规划先行、有序开发、留有余地的要求,完成城市大框架构建。优化发展中心城区,推进即墨、胶州、胶南城市综合功能的提升和发展,扩大平度、莱西城区空间规模。开发琅琊、鳌山、仰口、田横等滨海组团,加快温泉、凤凰岛等旅游度假区建设步伐。高标准完成滨海公路、海湾大桥工程,海底隧道及部分配套工程争取完工。加快国际空港建设,形成飞机维修中心、国际货运中心、飞行航运中心、生活服务中心。开工建设城市轨道交通,完善城市客运公共交通体系,加大水、电、气、暖、通讯等公用设施建设力度,加强后备水源储备地建设,提升城市综合承载力。保护好历史优秀建筑和街区,延续城市文脉。加快旧城改造和城市绿化建设,不断改善城区面貌。深化城市管理体制综合改革,推进城市管理现代化。全面实施《青岛奥运行动规划》,举办一届有特色、高水平的奥运会帆船比赛。规划建设邮轮母港。

　　(五)加大改革开放力度,建立更具活力更加开放的经济体制。实施改革攻坚,加快形成有利于转变经济增长方式、促进全面协调可持续发展的机制。深化行政管理体制改革,进一步转变政府职能,提高行政效率,建设服务型、法治型政府和高绩效机关。加快国有大型企业股份制改革,基本完成国有经济布局结构调整和国有企业改制任务。建立健全国有资本经营预算制度和国有资产管理监督营运体系,确保国有资产保值增值。加强政府投资管理,完善投资决策责任制度。创新财源建设机制,大力发展财源经济,实现财政收入稳定增长。推进现代市场体系建设,积极发展要素市场,整顿规范市场秩序,建立完善社会信用体系。鼓励民营企业开展技术创新、产品开发和争创名牌,向专业化、规模化、集约化、集群化发展。

　　提高对外开放水平和质量。积极吸引外资投向产业集群、高新技术产业、现代服务业和城市基础设施,鼓励跨国公司在青设立地区总部、研发中心、采购中心。优化出口商品结构,提高加工贸易产业层次和加工深度。支持有条件的企业“走出去”。拓宽国内经济合作渠道,吸引更多的国家重点企业和知名民营企业来青投资。继续做好对口帮扶菏泽曹县和支援贫困地区工作。

　　(六)坚持以人为本,努力构建社会主义和谐社会。强化政府促进就业的公共服务职能,完善城乡统筹促进就业工作机制,加快发展就业经济。健全与经济发展相适应的社会保障体系,扩大保障覆盖面,切实做好困难群体社会救助。大力发展富民经济,着力提高低收入者收入水平,扩大中等收入者比重,逐步提高最低生活保障和最低工资标准。加快住房建设,搞好配套设施,不断改善居民居住条件。强化政府对义务教育的保障责任,完善现代国民教育体系和终身教育体系,建设学习型城市。加强人力资源能力建设,加大人才引进和培养力度,改善人才环境,构筑区域性人才高地。坚持文化事业和文化产业协调发展,形成覆盖全社会的公共文化服务网络。以打造“帆船之都”、“影视之城”、“音乐之岛”为着力点,加快建设现代文化名城。大力开展全民健身运动,提高竞技运动水平。完善公共卫生和医疗服务体系,提高疾病预防控制和医疗救治服务能力,深化医疗卫生体制改革,逐步解决城乡群众“看病难、看病贵”问题。加强人口和计划生育机制建设,稳定低生育水平,提高人口素质。贯彻男女平等基本国策。加快发展老龄事业。

　　推进“平安青岛”和“文明青岛”建设。加强社会治安综合治理,健全公共安全体系和突发公共事件应急体系,认真落实安全生产责任制。强化维护社会稳定的领导机制和工作机制,进一步增强处置各类突发公共事件的能力和解决人民内部矛盾的能力。做好奥帆赛安全保卫工作。加强基层民主建设,继续推进政务公开,促进司法公正,维护社会正义。深化“双学三创”活动,弘扬城市精神,塑造更加和谐文明的人文环境。建立健全创建文明城市工作长效机制,深入开展群众性精神文明创建活动,加强社会公德、职业道德和家庭美德建设,扩大注册志愿者规模,提高公民素质和城乡文明程度。繁荣发展哲学社会科学。巩固军政军民团结,促进经济建设和国防建设协调发展。

　　各位代表!全心全意为人民服务是人民政府的宗旨,推动经济发展、改善人民生活始终是我们的中心任务。我们一定坚持发展为了人民,发展依靠人民,发展成果由人民共享,把解决好民生问题放到突出的位置,立足于现实条件和财力可能,更加重视提高城乡居民的生活质量和水平,更加重视解决关系人民群众切身利益的问题,更加重视维护和实现社会公平,决不辜负全市人民的信任和期望。

　　三、2006年的主要工作

　　今年,是全面实施“十一五”规划的起步之年,做好全年的各项工作,为“十一五”发展开好局,意义十分重大。我们要按照中央和省经济工作会议的部署,坚持科学发展观,认真落实宏观调控政策,着力加快改革开放,着力增强自主创新能力,着力推进经济结构调整和增长方式转变,着力提高经济增长质量和效益,实现更快更好发展,促进和谐社会建设。经济社会发展的主要预期目标是:全市生产总值增长14%,地方财政一般预算收入增长14%,全社会固定资产投资增长25%,社会消费品零售总额增长14.5%,城市居民人均可支配收入和农民人均纯收入均增长10%,城镇登记失业率控制在4%以内,居民消费价格指数控制在103左右。

　　(一)加快推进重点产业项目建设

　　按照“十一五”产业结构调整和集群发展的要求,今年市政府确定产业重点项目95个,总投资1100亿元。其中大炼油、中船重工造修船基地、山东液化天然气接收终端(LNG)等过50亿元项目4个,招商局国际集装箱码头、即发东丽、海洋石油工程基地等10-50亿元项目25个。年内新开工项目20个,完工项目20个,完成投资230亿元以上。出台推进重点项目、产业集群零部件配套实施意见和落户产业园政策,增加产业集群发展专项资金。加快建设汽车产业配套园区,形成10万辆微型乘用车的配套能力。推出100个家电配套产品目录,全年确保20个项目投资建设。前湾港南岸作业区全面开工,董家口工业区按规划尽快启动,黄岛油港区加快建设,鳌山港区、四方港区加大招商力度。推进中船重工“六厂四所”建设,做好吸引深海钻井平台和大马力低速柴油机项目在青投资的工作。争取国家批准建设石化产业基地。

　　(二)着力增强自主创新能力

　　以国家创新体系企业研发中心试点为突破口,加强企业研发中心和重点实验室建设。推进高新区扩区工作,调整高新区布局,理顺领导体制和工作机制。加快构建青岛海洋科学数据中心、基础研究资源共享服务平台、中小企业公共研发服务平台和高新技术创新服务中心。抓好国家海洋科学研究中心建设,培育3-4个海洋高新技术产业化示范基地。加强人才资源的开发利用,启动“222”引才工程,优化高层次人才创业环境。做好知识产权保护和技术标准工作,研究出台支持自主创新的相关政策。

　　集中力量在电子信息、新材料、新能源、生物技术、节能环保等重点领域开展科研攻关。以信息家电核心芯片及嵌入式软件为重点,组织集成电路专项技术攻关,信息家电芯片自主研发迈出新步伐;以制造业信息化、重大装备制造业技术研发为重点,突出关键技术的引进消化吸收,建设具有国内先进水平的基于无线射频识别的数字生产线;以太阳能等新能源技术开发为重点,开展光电子材料和半导体照明技术产业化研究;以循环经济关键技术研发为重点,在海水淡化利用、风力发电、工业废弃物处理和白泥治理等方面取得突破。积极推进节约型社会建设,抓好钢铁、电力、化工等重点行业和重点企业的节能、节水工作。全市高新技术产业产值突破2000亿元。

　　(三)努力推动农村经济社会协调发展

　　全面贯彻落实中央农村工作会议精神,做好“三农”工作,为建设社会主义新农村开好局。以发展现代农业为目标,加大农业结构调整力度,实施优质粮食产业工程,集中扶持四个100万亩优势农产品生产基地,突出发展高效渔业和畜牧业,切实加强重大动物疫病预防与控制。积极推进农业产业化经营,促进骨干龙头企业做大做强,抓好10处农产品产地批发市场升级改造,培育市级示范合作经济组织30个。继续实施城乡统筹、互动发展战略,壮大郊区经济实力,集中力量推动平度、莱西两市加快工业化进程。加强农村基础设施和社会事业建设。实施大中型水库上游和大沽河生态保护、中小水库除险加固等水利项目。按照新的村镇总体规划要求,抓好重点镇和小康示范村建设。加快推进“村村通柏油路”工程,今年实现“村村通自来水”。两年内全部免除农村义务教育学杂费。进一步完善农村卫生服务体系,增加农村合作医疗财政补助。完成50个镇(街道)卫生院标准化建设。实施“文化家园”工程,完善农村公共文化服务体系,年内实现“镇镇有文化中心”的目标。建立五市被征地农民养老保险制度,提高农村低保标准和五保供养标准。实施1000户农村贫困残疾人安居工程。加快农村劳动力转移,大规模开展农村劳动力技能培训,拓宽农民增收渠道。稳定和完善各项支农政策,市财政对“三农”投入增长23.8%,达到11.8亿元。

　　推进农村综合改革。切实转变乡镇政府职能,进一步规范乡镇政府机构和事业单位设置。完善“以县为主”的农村义务教育管理体制,建立各级政府合理分担的义务教育经费保障机制。调整完善区市、乡镇财政体制,加大对困难镇村转移支付力度。全面落实土地延包政策,巩固农民减负成果。支持村集体经济股份合作制改造试点。建立农业信用担保体系,探索建立多样化的农业投入新机制。(四)切实提高对外开放的质量和效益

　　积极有效地利用外资。全年吸引外商直接投资33亿美元。围绕建设六大产业集群,着力抓好120个产业链项目的定向招商。加大重点园区建设与引资工作,抓住机遇,努力把开发区、高新区、保税区和出口加工区等建设成为精品园区。依托大企业继续做好“以强引强”、以龙头引配套工作。突出对日、韩、德、台等重点国家和地区招商,在引进台资项目和欧美中小企业合作发展方面迈出新步伐。努力促进现有外资项目增资扩产。扩大城市基础设施、公益设施对外开放,推动流亭国际机场对外合资合作,积极推进前湾港区外资集装箱码头等项目建设。加强国内招商工作,全年实际到位内资300亿元。着力优化以项目为重点的投资环境和以企业为重点的发展环境,进一步完善投诉处置、责任追究、案例通报等制度,严肃查处一批严重影响发展环境的行为,全力打造最佳的生态环境、人文环境、社会环境和法治环境,努力形成万商云集、万众创业的开放局面。

　　加快外贸增长方式转变。以产业结构调整促进对外贸易结构优化,努力扩大高附加值产品出口,高新技术和机电产品出口分别增长25%和20%以上。促进外贸公司由专业性向综合商社转变。推动新投产项目尽快形成出口增长点,扶持民营企业成为拉动出口的重要力量,鼓励出口企业拓展新兴国际市场。支持企业增加国内急需的能源、重要原材料、关键技术和重大设备的进口。建立健全应对国际贸易摩擦的工作机制,强化对企业的信息服务,引导企业善于运用国际通行规则处理贸易争端和保护自己。加快“大通关”和电子口岸建设,提高通关效率。推动企业更快更好地“走出去”,进一步利用好国际国内两个市场、两种资源。

　　(五)进一步加强城市规划建设管理

　　健全城市规划体系,强化规划先导作用。编制好滨海公路各沿线组团控制性规划、海洋功能区划和海洋环境保护规划、空港与临空经济区发展规划。重视城市设计工作,完成城市轨道交通规划和城市主要干道、街区的设计。

　　加快城市基础设施建设。推进海湾大桥、机场扩建二期、滨海公路南段、即墨至平度高速公路、青岛至莱芜高速公路、青岛至龙口高速公路等工程建设。滨海公路北段年内完工。基本完成奥运场馆建设,办好奥帆测试赛。争取海底隧道年内开工。做好轨道交通项目前期准备工作。加快华电青岛发电公司二期热电联产热网工程和浮山新区供热站等供热工程建设。开展建筑节能工作,全年完成节能建筑500万平方米。全面实施生态市建设规划,做好山头、公园、道路的绿化和综合整治,加强李村河下游河道治理,完成团岛中水厂、麦岛污水处理厂扩建、团岛流域和前海一线截污工程。全面建成国家环保模范城市群。

　　切实提高城市管理水平。认真落实城市管理权限属地化的各项政策,完善城管执法目标管理、执法保障等体系和公安城管联动机制。积极推进城管执法工作向重点镇、中心镇延伸。集中开展以户外广告、占路经营等为重点的综合整治行动,创建70条城管示范达标路。加强市政工程及道路施工的统一协调和管理。规范城中村改造。完善物业管理招投标,重点培育一批优秀物业管理企业,不断提高住宅小区物业管理水平。

　　(六)全面深化经济体制改革

　　贯彻实施《青岛市进一步深化经济体制改革的意见》。不断优化国有经济布局,深化国有大企业改革,推进国有资本向优势产业和知名品牌集中,增强国有资本带动作用。规范产权交易“招、拍、挂”工作,基本完成国有中小企业改制。组建行业托管中心,加快国有资产经营公司重组。基本完成上市公司股权分置改革任务。推进市政公用、交通、广电等领域经营性国有资产的监管体制和运营方式改革。完善国有资产监管业绩考核体系,抓紧建立国有资本经营预算制度。深化投融资体制改革,加快建立市场化的融资方式,提高资本运作能力。推行政府投资项目代建制。进一步完善公用事业价格形成机制,稳妥做好价格调整工作。推进劳动力、土地、技术、产权等要素市场建设,规范发展各类中介组织。大力发展财源经济,对重点税源企业实行激励政策,鼓励引进质量型、环保型和对地方财政贡献大的产业项目。

　　放手发展民营经济。认真落实促进民营企业发展的各项政策,在融资服务等方面取得新进展。鼓励和支持民营企业参与国企改革和重组。依托工业园区,扶持特色民营经济板块发展,为产业集群协作配套。设立民营及中小企业发展专项资金,重点扶持一批高成长性中小企业。新增营业收入过亿元企业30家。实现民营经济增加值增长19%,税收增长16%。

　　(七)积极推进服务业发展和扩大消费

　　围绕构建区域性金融中心,促进城市商业银行、农村信用社等地方法人金融机构发展,年内引进5-6家国内外金融保险机构。加快保税物流园区、空港物流园区建设,大力发展生产资料市场。推进极地海洋世界、现代艺术中心等项目建设,加强旅游市场管理和区域旅游合作,加大“帆船之都”城市品牌推介力度,旅游总收入突破300亿元。建成启用国际会展中心二期。精心组织好第四届APEC中小企业技术交流暨展览会、2006中国国际消费电子博览会等重大展会。制定楼宇经济发展规划,促进总部经济健康发展,打造2-3个高端商业服务集聚区。

　　着力扩大消费需求。不断改善消费环境,提高服务质量,满足群众多样化的消费需要。加强流通基础设施建设,大力发展连锁超市、便利店、网上购物等新型流通业态,加快17条特色商业街建设,尽快形成品牌效应。继续扩大住房、汽车、信息、健身等消费热点,扩大消费信贷。把增加居民消费特别是农民消费作为扩大消费的重点,实施“万村千乡”市场工程,完善农村商业流通服务设施。

　　(八)努力促进社会更加文明和谐

　　着力解决人民群众最关心、最直接、最现实的利益问题。加大为群众办实事力度,切实抓好市政府为改善群众生产、生活条件要办的10件实事。健全公共就业服务体系,统筹促进城乡就业。继续实施积极的就业政策,努力拓宽就业渠道,增加就业岗位。启动“现代技能型人才培养工程”,推进公共实训基地建设,建立政府扶助、社会参与的职业技能培训体系,建成高级技工学校新校区。全年实现城乡就业20万人。进一步完善社会保障体系。加强社会保险扩面征缴,完善城镇职工基本养老、基本医疗、失业、生育、工伤保险制度。建立社会保险储备金制度,拓宽社会保障筹资渠道。加快城乡特殊困难群众社会救助体系建设,发展社会福利和慈善事业。继续扩大经济适用住房和普通商品住房建设规模,规范销售管理。加快小型停车场和街头停车场建设,对部分车流量大的交通节点实施改造。加大小街小巷和破旧楼院的整治力度。完成仙家寨水厂改建和7万户自来水“一户一表”的改造。

　　加快发展社会事业。集中建设一批“优质、特色”初中学校,深入推进基础教育课程改革,完成市内四区义务教育管理体制属地化调整,提高职业技术教育水平。打造城市文化品牌,加强公共文化设施建设,改扩建60个社区文化中心,丰富群众精神文化生活。发展壮大文化产业,积极推进文化领域投融资体制改革,完善文化产业政策,大力发展新闻出版、广播影视、演艺娱乐、网络传媒等产业,培育和规范文化市场体系。加强对外文化交流与合作。进一步健全城市公共卫生服务体系,推进60个社区卫生服务中心(站)的标准化建设,大力规范医疗服务秩序。全面实施《奥运争光计划》,积极开展群众性体育活动。完善人口与计划生育新机制,积极推进优生优育工作。认真实施妇女儿童发展纲要,加强未成年人权益保护,加快老龄事业发展。做好双拥工作,进一步提高民兵预备役建设水平。促进民族、宗教、侨务、对台、红十字、档案、史志等工作全面发展。

　　按照“创建全国最安全、最稳定、最和谐城市之一”的要求,完善严打长效机制和动态治安防控体系,开展“基层基础年”建设活动,保障社会平安稳定。完善城市应急体系,确保人民群众生命财产安全。严格落实安全生产责任制和各项管理措施,加大执法力度和责任追究力度,突出抓好危险化学品、火灾、道路交通和重大气象灾害等专项整治和隐患排查,努力杜绝重大安全事故发生。完善食品药品安全保障体系,加强重点商品、重点领域和重点环节的综合监管。做好社会矛盾纠纷排查调处工作,进一步畅通信访渠道,依法维护信访者权益,引导群众以合法方式解决利益矛盾。贯彻落实《公民道德建设实施纲要》深化城市精神市民践行活动,切实加强未成年人思想道德教育。扩大创建文明城市成果,广泛开展创建文明社区、文明村镇、文明单位活动,提高市民文明素质。

　　(九)切实加强政府自身改革和建设

　　深化行政管理体制改革,加快政府职能转变。继续推进政企分开、政资分开、政事分开、政府与市场中介组织分开,明确规范政府职能定位,在搞好经济调节和市场监管的同时,更加注重履行社会管理和公共服务职能,着力为市场主体创造公平竞争环境。进一步完善行政管理决策机制,健全对涉及经济社会发展全局的重大事项决策的协商和协调机制,健全对专业性、技术性较强的重大事项决策的专家论证、技术咨询、决策评估制度,健全对与群众利益密切相关的重大事项决策的公示和听证制度,推进行政管理决策科学化、民主化。完善对行政管理权力的监督机制,强化对决策和执行等环节的监督,建立体现科学发展观和正确政绩观要求的干部实绩考核评价制度。推进事业单位分类改革,逐步规范聘用制管理。继续推行综合行政执法试点工作。建立健全财政资金绩效管理机制,提高财政资金使用效益。加强民主法制建设,提高依法行政水平。认真贯彻市委《关于进一步加强人大工作的意见》,严格执行人大及其常委会的各项决议决定,完善接受监督的工作机制。积极支持人民政协履行政治协商、民主监督、参政议政职能,主动听取各民主党派、工商联、无党派人士及各人民团体的意见和建议。切实做好代表议案、建议和政协提案办理工作。各级政府要主动接受社会公众监督和舆论监督。认真贯彻《行政许可法》和《全面推进依法行政实施纲要》,按照法定的权限和程序行使权力。推进行政执法评议考核工作,严格执行行政执法责任制和行政过错责任追究制。深入开展“市民月”活动,继续办好“行风在线”,广泛倾听各方面意见。

　　以“创建高绩效机关、做人民满意的公务员”为目标,切实加强政府机关和公务员队伍建设。所有公务员特别是各级领导干部都要牢记“两个务必”,努力提高贯彻科学发展观的能力、驾驭全局的能力、处理各种利益关系的能力和务实创新的能力。认真贯彻实施《公务员法》,培育和弘扬公务员精神,加强公务员管理,进一步转变作风。以提升能力素质为重点,开展大规模公务员培训,不断优化结构,建设一支政治坚定、业务精湛、作风过硬、人民满意的公务员队伍。建立高效政务信息化平台,积极推行网上审批,加快政府工作流程再造,减少环节,优化程序,提高效率,增强行政透明度。全面落实首问责任制、服务承诺制、限时办理制等制度,提高政府执行力和公信力。加强执法监察和效能监察,充分发挥审计在维护财经秩序、促进依法行政、加强廉政建设等方面的作用。深入开展党风廉政建设和反腐败斗争,进一步完善教育、制度、监督并重的惩治和预防腐败体系。强化对党员干部特别是领导干部作风的监督检查,大力倡导谦虚谨慎、艰苦奋斗的优良作风,提高廉洁从政意识和拒腐防变能力。

　　各位代表!新的一年已经开始,面对新时期新目标新使命,我们深感任重道远,充满必胜信心。让我们紧密团结在以胡锦涛同志为总书记的党中央周围,高举邓小平理论和“三个代表”重要思想伟大旗帜,全面落实科学发展观,在中共青岛市委的正确领导下,进一步解放思想,与时俱进,扎实工作,全面完成“十一五”规划确定的各项任务,为把青岛建设成为全国重点中心城市和世界知名特色城市而努力奋斗!
